# Elevated carbonic anhydrase-1 in the aqueous humor in diabetic macular edema: associations between inflammatory cytokines and retinal vascular dysfunction

**DOI:** 10.3389/fmed.2026.1764980

**Published:** 2026-04-02

**Authors:** Jie Liu, Jingxin Zeng, Pengfei Ren, Xiao Ke, Fang Lu, Xiaoshuang Jiang

**Affiliations:** 1 Center for High Altitude Medicine, West China Hospital, Sichuan University, Chengdu, China; 2 Department of Ophthalmology, West China Hospital, Sichuan University, Chengdu, China; 3 Chengdu Kanghong Pharmaceutical Group Co Ltd., Chengdu, China

**Keywords:** aqueous humor, carbonic anhydrase-1, cytokine, diabetic macular edema, diabetic retinopathy

## Abstract

**Purpose:**

This study aimed to investigate the expression of carbonic anhydrase-1 (CA-1) in the aqueous humor of patients with diabetic macular edema (DME) and to explore its correlations with other cytokines and macular structure in a real-world setting.

**Methods:**

This was a retrospective cross-sectional study. A total of 74 patients were enrolled in the DME group, and 30 patients were included in the control group. Aqueous samples were collected before intravitreal injections or relevant surgical procedures. The concentrations of CA-1, prekallikrein (PK), intercellular adhesion molecule-1 (ICAM-1), vascular intercellular adhesion molecule-1 (VCAM-1), monocyte chemoattractant protein-1 (MCP-1), vascular endothelial growth factor (VEGF), interleukin-6 (IL-6), and interleukin-8 (IL-8) were measured using a cytometric bead array (CBA). Retinal structural biomarkers were examined using optical coherence tomography (OCT) and fundus photography.

**Results:**

The levels of CA-1 in the aqueous humor were markedly elevated in patients with DME compared to the control group. The DME group showed a significantly higher geometric mean CA-1 level than the control group (geometric mean ratio = 10.65, 95% CI: 3.64–31.14, *p* < 0.001). Receiver-operating characteristic curve analyses (AUC = 0.89; 95% CI: 0.871–0.981, *p* < 0.001) revealed that CA-1 has a strong discriminatory ability for distinguishing DME patients. In addition, other cytokines (PK, ICAM-1, VCAM-1, and IL-8) in the aqueous humor were elevated in the DME group compared to the control group (*p* < 0.05). In the DME group, CA-1 levels exhibited positive correlations with PK, ICAM-1, VCAM-1, MCP-1, VEGF, IL-6, and IL-8 (*r* = 0.306, 0.551, 0.347, 0.589, 0.349, 0.248, and 0.538, respectively; all *p* < 0.05). Conversely, CA-1 levels showed a negative correlation with retinal hemorrhage (*r* = −0.330, *p* = 0.016) and did not show significant association with other macular structural parameters.

**Conclusion:**

In DME patients, CA-1 levels were significantly elevated and showed associations with PK, ICAM-1, VCAM-1, VEGF, MCP-1, IL-6, and IL-8. These findings suggest that higher CA-1 levels are associated with increased retinal vascular permeability and inflammation in patients with DME.

**Clinical trial registration:**

https://www.chictr.org.cn/index.html, identifier 268319.

## 1 Introduction

Diabetic retinopathy (DR) is the most common microvascular complication of diabetes mellitus, with diabetic macular edema (DME) being the leading cause of vision loss in the working-age population globally (1). The global prevalence of diabetes mellitus is approximately 11.11% (2). Among patients with diabetes, the prevalence of DR is 22.27%. The global prevalence of DME among people with diabetes is approximately 4.07% (3). The rising prevalence of diabetes worldwide has resulted in a growing epidemic of DR, burdening healthcare systems and significantly impairing the quality of life for individuals affected by vision impairment (3). Chronic hyperglycemia in diabetic patients elevates vascular endothelial growth factor (VEGF) levels, leading to increased retinal vascular permeability. Furthermore, inflammatory factors contribute to leukocyte stasis and vascular impairment through inflammatory responses, playing a critical role in the progression of DR (4). Despite the established role of vascular and inflammatory factors in DR, additional mechanisms are also implicated, underscoring the complexity of the disease. Current treatments for DME primarily involve intravitreal injections of anti-VEGF agents or corticosteroids (5). While anti-VEGF therapies are the cornerstone of treatment, a subset of patients fails to exhibit a significant therapeutic response. Furthermore, the effects of anti-VEGF treatments diminish over time, necessitating repeated injections, which impose a substantial burden on patients. Steroid injections, while effective, are associated with side effects such as increased intraocular pressure and the development of cataracts over time. Consequently, the treatment of DME remains a challenge, as current therapies do not fully address all aspects of the condition. Further exploration of additional therapeutic pathways is needed for DME patients who exhibit inadequate responses to anti-VEGF or corticosteroid therapies. Additionally, there is a need to improve treatment efficacy and reduce frequency through the use of combination therapies.

Carbonic anhydrase-1 (CA-1) is a metalloenzyme that catalyzes the reversible hydration of CO₂ into bicarbonate ions and protons. CA-1, a cytoplasmic isoenzyme, is found in the red blood cells, gastrointestinal tract, vascular smooth muscle cells, and vascular endothelial cells, typically in a low-activity state (6). It plays a role in regulating vasomotion. Research indicates that CA-1 increases retinal vascular permeability through the kallikrein–kinin system (KKS) and exerts a synergistic effect with VEGF on retinal permeability (7). Notably, CA-1 inhibitors have been shown to dilate retinal blood vessels, enhance oxygen tension, and reduce vascular permeability, highlighting their potential therapeutic value (8–10).

This study examined changes in CA-1 levels in the aqueous humor of patients with DME and investigated their correlations with other cytokines and macular structure, aiming to provide hypothesis-generating insights into the exploration of diverse treatment strategies for DME.

## 2 Materials and methods

### 2.1 Ethics approval

This research process was performed in accordance with the Declaration of Helsinki and received approval from the Institutional Review Board of West China Hospital, Sichuan University (approval no.2023-1,375). Informed consent was obtained from all participants.

### 2.2 Patients and samples

This cross-sectional study was conducted at West China Hospital between 1 June 2022, and 30 December 2023. A total of 104 patients who visited the hospital were enrolled, including those with DME, cataracts, idiopathic macular holes, or epiretinal membranes. Patients were categorized into DME and control groups. Treated DME patients received anti-VEGF therapy (ranibizumab, aflibercept, or conbercept) with a regimen of one to three injections. The time from the last injection to the collection of samples was at least 1 month. No corticosteroid treatment was administered. The exclusion criteria included a history of ocular diseases or surgeries unrelated to the studied conditions or major systemic diseases.

**Figure 1:**
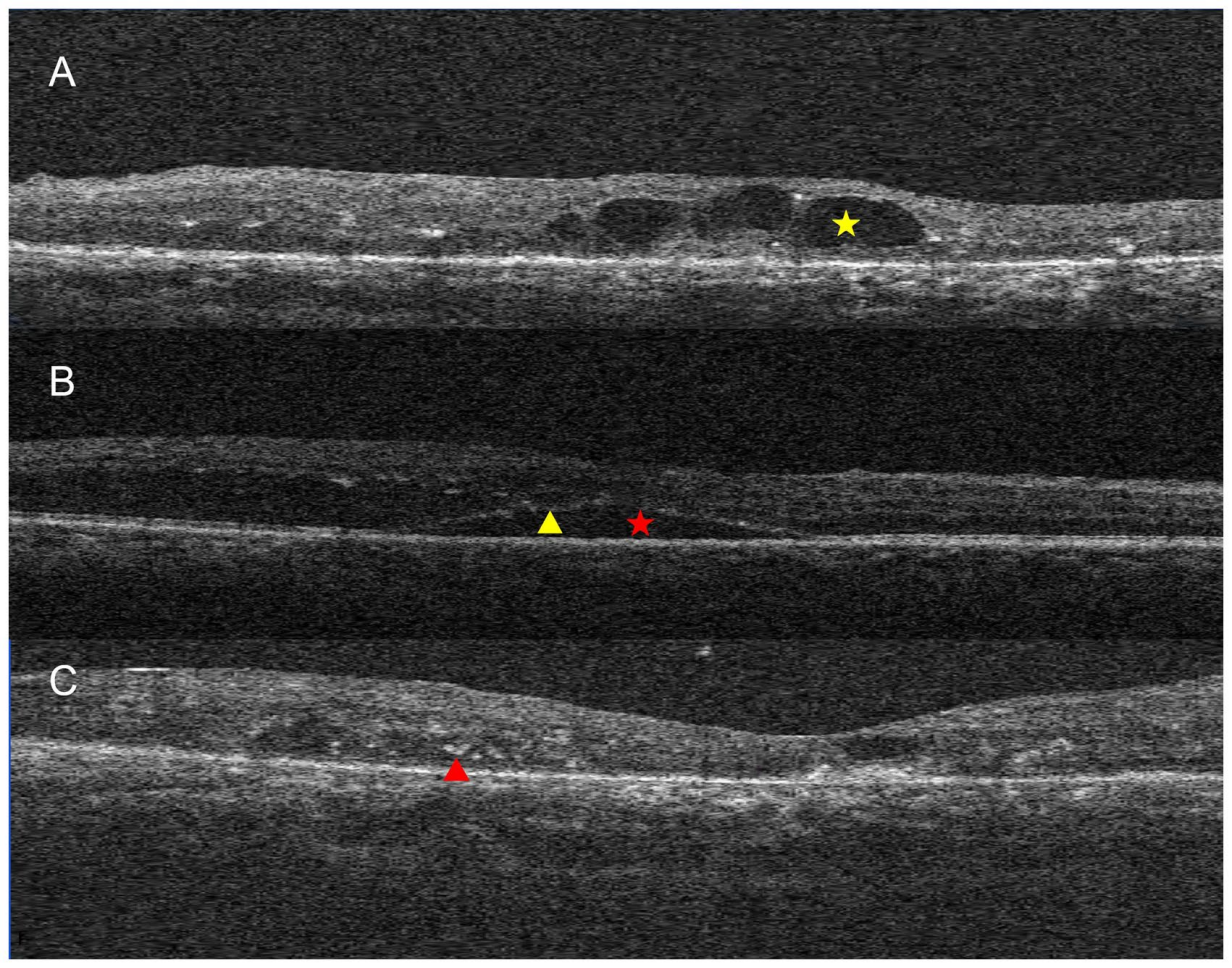
Examples of OCT-associated items. **(A)** Macular cystoid edema (marked by a yellow asterisk); **(B)** subretinal fluid (marked by a red asterisk), ellipsoid zone disruption (marked by a yellow triangle); **(C)** hyperreflective foci (marked by a red triangle).

Demographic data, including age and gender, were collected from all participants. Each patient underwent a comprehensive ophthalmic examination, which included visual acuity testing, slit-lamp evaluation, dilated fundus examination, fundus photography, and optical coherence tomography (OCT) (Zeiss Cirrus HD-OCT 6000, CA, USA). OCT data on central retinal thickness (CRT), subretinal fluid (SRF), cystoid macular edema (CME), retinal hemorrhage, ellipsoid zone (EZ) disruption, and hyperreflective foci (HRF) were carefully analyzed (Figure 1). These macular structural changes were analyzed by graders (JL and JXZ). In cases where there were discrepancies between their assessments, a third researcher (XSJ) was consulted to make a final determination. Detailed grading criteria are provided below: CRT was defined as the distance from the internal limiting membrane to the retinal pigment epithelium at the foveal center. CME was defined as the presence of intraretinal hyporeflective cystic spaces in the macula, and SRF was defined as a hyporeflective space between the neurosensory retina and the retinal pigment epithelium. Retinal hemorrhage was identified using OCT and color fundus photography as hyperreflective or shadowing lesions within the retinal layers. Disruption of the EZ was characterized by a break, irregularity, or loss of the normal hyperreflective EZ band at the fovea. HRF were defined as discrete, punctate, hyperreflective lesions within the retinal layers, distinct from the retinal vessels. CRT was recorded as a continuous variable, while SRF, CME, retinal hemorrhage, EZ disruption, and HRF were recorded as categorical variables, noted as either “present” or “absent” for statistical analysis.

Aqueous humor samples were collected before initiating anti-VEGF treatment or performing any ocular surgeries. Approximately 0.1 mL of the aqueous humor was obtained via limbal puncture using a 30-gauge insulin syringe needle. The aqueous humor samples were collected and stored at −80 °C with fewer than three freeze–thaw cycles until analysis. All samples were processed in a single batch to minimize batch effects. Cytokine levels were measured using a commercial detection platform; the limits of detection (LOD) and lower limits of quantification were provided by the manufacturer. Intra-assay and inter-assay coefficients of variation were within acceptable ranges, with all samples tested in duplicate. For statistical analysis, concentrations below the LOD were replaced with LOD/2. The concentrations of CA-1, prekallikrein (PK), intercellular adhesion molecule-1 (ICAM-1), vascular intercellular adhesion molecule-1 (VCAM-1), monocyte chemoattractant protein-1 (MCP-1), VEGF, interleukin-6 (IL-6), and interleukin-8 (IL-8) were measured using a cytometric bead array following the manufacturer’s protocol. Data acquisition was performed using a BD FACSCelesta™ flow cytometer (BD, CA, USA), and the data were analyzed using FCAP Array™ software (version 3.0) (BD, CA, USA). Standard curves were generated based on the reference concentrations provided in the kit, and cytokine concentrations were calculated accordingly.

### 2.3 Statistical analysis

All analyses were conducted using SPSS software (version 25.0). Categorical variables were presented as absolute frequencies and percentages, while quantitative variables were described using means, standard deviations, medians, and interquartile ranges. Normality was evaluated using the Shapiro–Wilk test. Comparisons were performed using the Student’s *t*-test, Mann–Whitney U-test, and chi-squared test. Correlations were assessed using Spearman’s correlation analysis. A Gamma generalized linear model with log link was used for independent association analysis. Receiver-operating characteristic (ROC) curve analysis was performed to evaluate discriminatory ability.

## 3 Results

### 3.1 Clinical characteristics

A total of 104 participants were included in this study. Demographic characteristics showed that patients with DME were significantly younger than those in the control group (*p* < 0.05). However, no significant differences were observed in gender distribution (*p* = 0.349) or visual acuity (*p* = 0.364) between the two groups. OCT imaging revealed distinct phenotypic differences between the groups. Quantitative assessments showed that the median CRT in the DME group was 291 (254, 361) μm, which was higher than 234 (217, 251) μm in the control group (*p* < 0.05). SRF, CME, retinal hemorrhage, and HRF were detected in 20, 67, 66, and 84% of DME patients, respectively, but none were found in the control group. Similarly, EZ disruption was frequent in DME patients (84%) but rare in controls (30%) (Table 1). After adjusting for age, further comparisons revealed that levels of CA-1, PK, ICAM-1, VCAM-1, and IL-8 remained significantly higher in the DME group compared to the control group (all *p* < 0.05). No statistically significant difference was observed in VEGF, MCP-1, or IL-6 levels between the two groups (*p* = 0.656, 0.628, and 0.239, respectively) (Table 2). VEGF levels were significantly higher in treatment-naïve DME patients compared to treated DME patients (*p* < 0.001; Supplementary Table 1). However, no significant differences in macular structure were observed between treatment-naïve and treated DME patients (Supplementary Table 2).

**Table 1:** Patient characteristics.

Characteristics	DME group (*n* = 74)	Control group (*n* = 30)	*p*-value
Age, years, Median (IQR)	57 (53, 61)	67 (58.75, 70)	<0.001*
Gender			0.349
Men	33 (46.5%)	13 (43.3%)	
Women	38 (53.5%)	17 (56.7%)	
LogMAR, Median(IQR)	0.60 (0.30, 1.40)	0.95 (0.50, 1.475)	0.364
Central retinal thickness, Median (IQR)	291 (254, 361)	234 (217, 251)	<0.05*
Subretinal fluid	20%	0	<0.001^#^
Cystoid macular edema	67%	0	<0.001^#^
Retinal hemorrhage	66%	0	<0.001^#^
Ellipsoid zone disruption	84%	30%	<0.001^#^
Hyperreflective foci	84%	0	<0.001^#^

IQR, interquartile range; statistically significant at *p* < 0.05. *Mann–Whitney U-test; ^#^chi-square test.

**Table 2:** Cytokine levels between DME and control groups.

Cytokines [median (IQR), pg./mL]	DME group (*n* = 74)	Control group (*n* = 30)	*p*-value
CA-1	4644.37 (2168.92, 76444.3551)	487.70 (190.37, 9297.74)	<0.001*
PK	63178.54 (39221.31, 97727.76)	11025.60 (7398.88, 26937.95)	<0.001*
ICAM-1	1246.30 (731.17, 1880.82)	107.35 (0, 432.93)	0.008*
VCAM-1	5917.14 (3337.51, 10529.66)	1996.85 (1267.16, 3997.11)	<0.001*
VEGF	32.80 (5.60, 109.93)	44.72 (22.76, 55.33)	0.656
MCP-1	908.72 (672.11, 1374.46)	510.02 (429.00, 639.67)	0.628
IL-6	45473.52 (11889.12, 128359.51)	6960.77 (3770.03, 12113.38)	0.239
IL-8	31697.56 (12224.70, 54319.82)	4572.25 (2508.23, 7679.43)	0.007*

IQR, interquartile range; CA-1, carbonic anhydrase-1; PK, prekallikrein; ICAM-1, intercellular adhesion molecule-1; VCAM-1, vascular intercellular adhesion molecule-1; VEGF, vascular endothelial growth factor; MCP-1, monocyte chemoattractant protein-1; IL-6, interleukin-6; IL-8, interleukin-8; *Statistically significant after adjusting for age (*p* < 0.05).

### 3.2 Association between CA-1 and diabetic macular edema

A generalized linear model (GLM) with a gamma distribution and log link was constructed to evaluate the independent association between CA-1 levels and DME status. After adjusting for age, gender, and other cytokines as confounding factors, CA-1 levels were significantly higher in the DME group than in the control group (*B* = 2.366, 95% CI: 1.283–3.348; Wald *χ*^2^ = 18.677, *p* < 0.001), with a corresponding geometric mean ratio of 10.65 (95% CI: 3.64–31.14, *p* < 0.001). These findings indicate a strong positive association between CA-1 expression and DME in the present study population.

**Figure 2:**
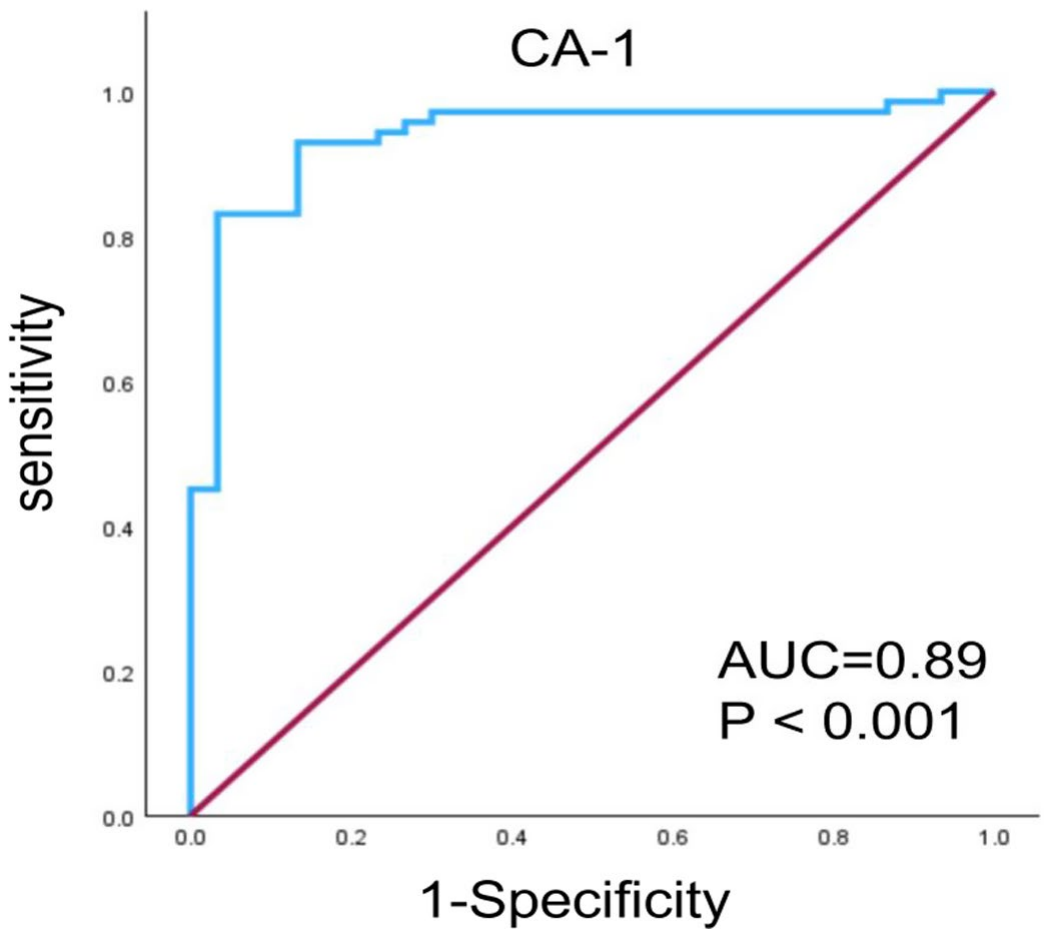
Receiver-operating characteristic (ROC) analysis for CA-1 to discriminate DME from controls after adjusting for age and gender with bootstrap internal validation. The AUC was 0.89 (95% CI: 0.871–0.981, *p* < 0.001). The optimal cutoff value of the predicted probability was 0.61, with a sensitivity of 86% and a specificity of 92%. The blue line represents the bootstrap-validated ROC curve, and the dotted red diagonal line represents a model with no discriminative ability.

We evaluated the discriminatory performance of CA-1 for DME using an age- and gender-adjusted multivariable logistic regression model. An ROC analysis was performed based on model-predicted probabilities, and internal validation was conducted via bootstrap resampling. The adjusted AUC was 0.89 (95% CI: 0.871–0.981, *p* < 0.001). According to the Youden index, the optimal cutoff value for the predicted probability was 0.61, with a sensitivity of 86% and a specificity of 92%. These results suggest that CA-1 exhibits strong discriminatory performance for DME (Figure 2).

### 3.3 Relationships between CA-1 and both other cytokines and macular structural parameters

In the DME group, CA-1 was positively correlated with PK, ICAM-1, VCAM-1, VEGF, MCP-1, IL-6, and IL-8 (*r* = 0.306, 0.551, 0.347, 0.349, 0.589, 0.248, and 0.538, respectively; all *p* < 0.05) (Table 3). Additionally, CA-1 levels in the DME group were negatively correlated with retinal hemorrhage (*r* = −0.330, *p* = 0.016), whereas no significant correlations were observed with other macular structural parameters (Table 4).

**Table 3:** Correlation between CA-1 and other cytokines in DME patients.

Cytokines	PK	ICAM-1	VCAM-1	VEGF	MCP-1	IL-6	IL-8
*r*	0.306	0.551	0.347	0.349	0.589	0.248	0.538
*p*-value	0.008*	<0.001*	0.002*	0.002*	<0.001*	0.042*	<0.001*

*Statistically significant *p*-value (<0.05).

**Table 4:** Correlation between CA-1 and macular structure in DME patients.

OCT biomarkers	CRT	SRF	CME	Hemorrhage	EZ disruption	HRF
*r*	−0.051	−0.112	−0.005	−0.330	−0.135	0.229
*p*-value	0.710	0.422	0.972	0.016*	0.332	0.095

*Statistically significant *p*-value (<0.05).

## 4 Discussion

Multiple cytokines are implicated in the pathogenesis of diabetic retinopathy, making its treatment challenging. CA-1 is expressed in various parts of the human eye, including corneal endothelial cells, lens cells, the choroid, and retinal cells (11). In our study, we also identified CA-1 in the aqueous humor. A study conducted on animals demonstrated that injecting CA-1 into the vitreous cavity of rats increased retinal vascular permeability, an effect that was effectively mitigated by the administration of acetazolamide or methazolamide (7). Our findings revealed that CA-1 levels in the aqueous humor of DME patients were significantly higher than those in the control group. Generalized linear model analysis revealed a higher geometric mean CA-1 level in the DME group than in the control group. The ROC curve analysis indicated that CA-1 could discriminate patients with DME from controls in this study. Previous studies have demonstrated that the concentration of CA-1 is elevated in the vitreous cavity of patients with DR. Our findings further demonstrate that CA-1 levels are also increased in the aqueous humor of DME patients.

The kallikrein–kinin system (KKS) operates through two serine protease zymogens—coagulation factor XII (FXII) and plasma PK—and their substrate, high-molecular-weight kininogen. Activating the intraocular KKS can lead to increased retinal vascular permeability, vasodilation, and retinal thickening (12–15). Studies have shown that elevated CA-1 levels in the vitreous humor can activate the KKS, while CA-1 inhibitors can reduce retinal vascular permeability by decreasing PK activity (16).

Our study found that PK concentrations were significantly higher in the DME group than in the control group and that CA-1 and PK were correlated in the DME group. We also observed elevated levels of IL-8, a chemokine with strong proinflammatory effects, ls in the DME group, which is consistent with findings from previous studies (17). CA-1 was found to be correlated with IL-8 in the DME group. ICAM-1, a key adhesion molecule in DR, facilitates leukocyte adhesion and migration, while VCAM-1, expressed on endothelial cells, increases with rising blood glucose levels (18). Our study revealed elevated levels of ICAM-1 and VCAM-1 in the DME group, and CA-1 was found to be correlated with both adhesion molecules. MCP-1, another chemokine secreted by Müller cells, recruits monocytes and macrophages and promotes retinal ischemia, angiogenesis, and inflammation, thereby aggravating retinal neuronal damage (19). IL-6 is a proinflammatory factor involved in immune regulation, vascular permeability, and angiogenesis (20). No significant differences were observed in MCP-1 and IL-6 levels between the DME and control groups in this study, which may be attributed to the relatively small sample size and individual heterogeneity in inflammatory status. Additionally, CA-1 levels were correlated with IL-6 and MCP-1 levels in DME patients. VEGF, a potent proangiogenic growth factor associated with neovascularization in DME, showed no significant difference in levels between the DME and control groups in our study. This lack of difference may be attributed to treatment interventions, as VEGF levels in the control group were lower than in the treatment-naïve group but higher than in the treated group. Furthermore, in the DME group, CA-1 was found to be correlated with VEGF levels. The CA-1-activated KKS may synergize with VEGF and inflammatory cytokines to promote the progression of diabetic retinopathy by altering retinal vascular permeability. Notably, this was an observational cross-sectional study. The observed association between CA-1 levels and inflammatory or vascular markers does not indicate causality but represents a hypothesis-generating finding, suggesting that CA-1 may be involved in biological pathways related to diabetic macular edema. Further prospective and experimental studies are needed to clarify the mechanistic role of CA-1 in disease progression.

Previous studies have confirmed that patients with DME exhibit characteristic OCT findings, including increased CRT, the presence of SRF and CME, alongside EZ disruption and HRF (21, 22). Our results consistently demonstrated similar OCT structural manifestations in DME patients. In our study, CA-1 was negatively correlated with retinal hemorrhage and showed no significant correlations with other macular structural biomarkers in the DME group. This seemingly paradoxical finding may reflect distinct pathophysiological states corresponding to DR severity. In diabetic retinopathy, ischemia and hypoxia disrupt the blood–retinal barrier, triggering CA-1 release into the intraocular fluid from stressed retinal cells. However, as DR progresses, retinal tissue shifts from a compensatory stress response to a state of functional exhaustion, thereby reducing CA-1 secretion and yielding the observed negative correlation. Our findings may reflect the challenges of real-world clinical settings. Poor patient compliance with medical recommendations and suboptimal management of underlying diseases are common complicating factors (23, 24). Furthermore, OCT scans were obtained before each new anti-VEGF injection, potentially revealing the effects of treatment waning over time.

This study has several limitations. First, participants were recruited from a prominent grade—a tertiary hospital in southwest China, where patients typically present with severe and complex conditions. Second, the relatively small sample size may limit the comprehensive assessment of clinical phenotypes and individual differences among DME patients, suggesting that weaker associations might not be detected due to insufficient statistical power. Third, the study focused only on the levels of the aqueous humor at a single time point, lacking long-term dynamic monitoring of these factors in diabetic patients.

In conclusion, our study demonstrated that the concentration of CA-1 in the aqueous humor of DME patients is significantly elevated and is correlated with PK, ICAM-1, VCAM-1, VEGF, MCP-1, IL-6, and IL-8. CA-1 may contribute to the progression of diabetic retinopathy by modulating retinal vascular permeability, synergizing with inflammatory responses and the VEGF pathway. However, causal relationships cannot be inferred. Further prospective and experimental studies are warranted to elucidate the biological role and potential clinical value of CA-1 in diabetic retinopathy.

## Data Availability

The raw data supporting the conclusions of this article will be made available by the authors, without undue reservation.

## References

[1] Meng Y Liu Y Duan R Liu B Lin Z Ma Y . Global, regional, and National Epidemiology of vision impairment due to diabetic retinopathy among working-age population, 1990-2021. J Diabetes. (2025) 17:e70121. doi: 10.1111/1753-0407.70121, 40660082 PMC12259346

[2] Genitsaridi I Salpea P Salim A Sajjadi SF Tomic D James S . 11th edition of the IDF diabetes atlas: global, regional, and national diabetes prevalence estimates for 2024 and projections for 2050. Lancet Diabetes Endocrinol. (2026) 14:149–56. doi: 10.1016/s2213-8587(25)00299-2, 41412135

[3] Teo ZL Tham YC Yu M Chee ML Rim TH Cheung N . Global prevalence of diabetic retinopathy and projection of burden through 2045: systematic review and Meta-analysis. Ophthalmology. (2021) 128:1580–91. doi: 10.1016/j.ophtha.2021.04.027, 33940045

[4] Wang W Lo ACY. Diabetic retinopathy: pathophysiology and treatments. Int J Mol Sci. (2018) 19:1816. doi: 10.3390/ijms19061816, 29925789 PMC6032159

[5] Tatsumi T. Current treatments for diabetic macular edema. Int J Mol Sci. (2023) 24:9591. doi: 10.3390/ijms24119591, 37298544 PMC10253534

[6] Berg JT Ramanathan S Gabrielli MG Swenson ER. Carbonic anhydrase in mammalian vascular smooth muscle. J Histochem Cytochem. (2004) 52:1101–6. doi: 10.1369/jhc.4A6266.2004 15258186

[7] Gao BB Clermont A Rook S Fonda SJ Srinivasan VJ Wojtkowski M . Extracellular carbonic anhydrase mediates hemorrhagic retinal and cerebral vascular permeability through prekallikrein activation. Nat Med. (2007) 13:181–8. doi: 10.1038/nm1534, 17259996

[8] Weiwei Z Hu R. Targeting carbonic anhydrase to treat diabetic retinopathy: emerging evidences and encouraging results. Biochem Biophys Res Commun. (2009) 390:368–71. doi: 10.1016/j.bbrc.2009.10.031, 19833100

[9] Pedersen DB Koch Jensen P la Cour M Bach Pedersen D Kiilgaard JF Eysteinsson T . Carbonic anhydrase inhibition increases retinal oxygen tension and dilates retinal vessels. Graefes Arch Clin Exp Ophthalmol. (2005) 243:163–8. doi: 10.1007/s00417-003-0817-3, 15742212

[10] Wolfensberger TJ. The role of carbonic anhydrase inhibitors in the management of macular edema. Doc Ophthalmol. (1999) 97:387–97. doi: 10.1023/a:1002143802926, 10896355

[11] Hallaj S Shalaby WS Sinha S Myers JS Razeghinejad R. Systemic carbonic anhydrase inhibitors in common ophthalmic diseases: a scoping review from a clinical standpoint. Curr Ophthalmol Rep. (2025) 13:9. doi: 10.1007/s40135-025-00332-x, 40642047 PMC12238189

[12] Abdulaal M Haddad NM Sun JK Silva PS. The role of plasma kallikrein-kinin pathway in the development of diabetic retinopathy: pathophysiology and therapeutic approaches. Semin Ophthalmol. (2016) 31:19–24. doi: 10.3109/08820538.2015.1114829 26959125

[13] Mantripragada S Wang SX Chilcote T Sinha S. Nonclinical safety and pharmacology of RZ402, a plasma kallikrein inhibitor, for the treatment of diabetic macular edema as a daily oral therapy. Invest Ophthalmol Vis Sci. (2020) 61:307

[14] Dugel PU Khanani AM Berger BB Patel S Fineman M Jaffe GJ . Phase 1 dose-escalation study of plasma kallikrein inhibitor THR-149 for the treatment of diabetic macular edema. Transl Vis Sci Technol. (2021) 10:28. doi: 10.1167/tvst.10.14.28, 34940810 PMC8711005

[15] Wisniewski P Gangnus T Burckhardt BB. Recent advances in the discovery and development of drugs targeting the kallikrein-kinin system. J Transl Med. (2024) 22:388. doi: 10.1186/s12967-024-05216-5, 38671481 PMC11046790

[16] Liu J Feener EP. Plasma kallikrein-kinin system and diabetic retinopathy. Biol Chem. (2013) 394:319–28. doi: 10.1515/hsz-2012-0316, 23362193 PMC4844060

[17] Mason RH Minaker SA Lahaie Luna G Bapat P Farahvash A Garg A . Changes in aqueous and vitreous inflammatory cytokine levels in nonproliferative diabetic retinopathy: systematic review and meta-analysis. Can J Ophthalmol. (2025) 60:e100–16. doi: 10.1016/j.jcjo.2024.05.031, 39043257

[18] Joy SS Siddiqui K. Molecular and pathophysiological mechanisms of diabetic retinopathy in relation to adhesion molecules. Curr Diabetes Rev. (2019) 15:363–71. doi: 10.2174/1573399814666181017103844, 30332969

[19] Taghavi Y Hassanshahi G Kounis NG Koniari I Khorramdelazad H. Monocyte chemoattractant protein-1 (MCP-1/CCL2) in diabetic retinopathy: latest evidence and clinical considerations. J Cell Commun Signal. (2019) 13:451–62. doi: 10.1007/s12079-018-00500-8, 30607767 PMC6946768

[20] Manda AR Lee LH Steinkerchner MS Sheng J Veach L Gangaputra S . Analysis of aqueous Interleukin-6 in diabetic retinopathy: a prospective, controlled trial of 328 eyes. Ophthalmol Retina. (2026) 10:81–7. doi: 10.1016/j.oret.2025.06.014, 40582698 PMC12260920

[21] Midena E Lupidi M Toto L Covello G Veritti D Pilotto E . AI-assisted OCT clinical phenotypes of diabetic macular edema: a large cohort clustering study. J Clin Med. (2025) 14:7893. doi: 10.3390/jcm14227893, 41302929 PMC12653931

[22] Okudan S Acar Duyan S Erdem A Bozkurt Oflaz A Turgut Ozturk B. Optical coherence tomography biomarkers predict the long-term restorative effect of early anti-VEGF treatment on diabetic macular edema. Life. (2025) 15:269. doi: 10.3390/life15020269, 40003678 PMC11857452

[23] Biechl AC Bhandari S Nguyen V Arnold JJ Young S Fraser-Bell S . Changes in real-world treatment patterns for diabetic macular oedema from 2009 to 2019 and 5-year outcomes: data from the fight retinal blindness! Registry. Clin Experiment Ophthalmol. (2020) 48:802–12. doi: 10.1111/ceo.13781, 32383527

[24] Holekamp NM Campbell J Almony A Ingraham H Marks S Chandwani H . Vision outcomes following anti-vascular endothelial growth factor treatment of diabetic macular edema in clinical practice. Am J Ophthalmol. (2018) 191:83–91. doi: 10.1016/j.ajo.2018.04.010, 29684329

