# Correction: Elevated carbonic anhydrase-1 in the aqueous humor in diabetic macular edema: associations between inflammatory cytokines and retinal vascular dysfunction

**DOI:** 10.3389/fmed.2026.1892721

**Published:** 2026-06-11

**Authors:** Jie Liu, Jingxin Zeng, Pengfei Ren, Xiao Ke, Fang Lu, Xiaoshuang Jiang

**Affiliations:** 1 Center for High Altitude Medicine, West China Hospital, Sichuan University, Chengdu, China; 2 Department of Ophthalmology, West China Hospital, Sichuan University, Chengdu, China; 3 Chengdu Kanghong Pharmaceutical Group Co Ltd., Chengdu, China

**Keywords:** aqueous humor, carbonic anhydrase-1, cytokine, diabetic macular edema, diabetic retinopathy

Affiliation “Center for High Altitude Medicine, West China Hospital, Sichuan University, Chengdu, China” was omitted from the paper. This affiliation has now been added for authors Jie Liu and Xiaoshuang Jiang.

An incorrect **Funding** statement was provided. The correct funding statement reads:

“The author(s) declared that financial support was received for this work and/or its publication. This study was supported by grant from 1·3·5 Project of Center for High Altitude Medicine, West China Hospital, Sichuan University (No. GYYX24011), the Natural Science Foundation of Sichuan Provincial Science and Technology Department (2023NSFSC1666) and China Health Promotion Foundation (312251372). The funders played no role in study design, data collection and analysis, decision to publish, or preparation of the manuscript.”

The original version of this article has been updated.

