# Supplementary material for: Elevated carbonic anhydrase-1 in the aqueous humor in diabetic macular edema: associations between inflammatory cytokines and retinal vascular dysfunction
Source: Front Med (Lausanne). 2026 Apr 2;13:1764980. doi: 10.3389/fmed.2026.1764980 (PMC13082990; doi:10.3389/fmed.2026.1764980)
Supplement: Supplementary file 1 [file Table_1.docx]

| Cytokine | Treatement naive (n=29) | Treated   (n=45) | Control  (n=30) | P value | | |
| --- | --- | --- | --- | --- | --- | --- |
|  |  |  |  | Treatement naive  VS Control | Treated  VS Control | Treatement naive  VS Treated |
| VEGF | 83.17（22.88，185.50） | 15.01（2.49，59.36） | 44.72（22.76，55.33） | P=0.025* | P=0.041* | P=0.001* |

Comparisons were performed among the treatment-naïve DME group, the treated DME group, and the control group.

| **Variables** | **Treatment-naive (n=29)** | **Treated(n=45)** | **P value** |
| --- | --- | --- | --- |
| CRT(um) | 305.13±59.78 | 284(242,372) | 0.561 |
| SRF(%) | (28%) | (70%) | 0.249 |
| CME(%) | (64%) | (68%) | 0.624 |
| RH(%) | (79%) | (58%) | 0.062 |
| EZ(%) | (80%) | (86%) | 0.514 |
| HRF(%) | (84%) | (86%) | 0.818 |

Comparison of macular structure between treatment-naïve and treated groups in DME patients.
